# Health behaviors, physical activity, and subjective well-being: rural–urban inequities among double first-class university graduate students in China

**DOI:** 10.3389/fpubh.2026.1874749

**Published:** 2026-07-21

**Authors:** Linqing Li, Guohua Zheng

**Affiliations:** 1 School of Economics and Management, Shanghai University of Sports, Shanghai, China; 2 School of Journalism and Communication, Shanghai University of Sports, Shanghai, China

**Keywords:** graduate students, insomnia, life satisfaction, physical activity, rural–urban disparity, subjective well-being

## Abstract

**Background:**

Graduate student well-being is an emerging public health priority, yet the role of pre-university structural background, particularly rural versus urban origin, remains poorly understood. This study examined whether rural–urban disparities in life satisfaction persist among students at an elite Chinese university, and to what extent health behaviors account for any observed gap.

**Methods:**

In a cross-sectional survey, 497 master’s and doctoral students at a Double First-Class university in eastern China completed the Satisfaction with Life Scale (SWLS), the IPAQ short-form physical activity questionnaire, and items on insomnia, sleep duration, and sociodemographic characteristics, including monthly disposable income. Two hierarchical linear regression models were constructed: Model 1 (sociodemographic variables only) and Model 2 (adding sleep, physical activity, and insomnia). Because Model 1 is a proper subset of Model 2, the attenuation of the rural-origin coefficient between model estimates the share of the gap associated with health behaviors.

**Results:**

After adjustment for sociodemographic covariates, rural-origin students scored 1.95 points lower on the SWLS than urban-origin peers (Model 1, *p* = 0.003). Adding health behaviors to the model reduced this gap by roughly half (*p* = 0.088). Insomnia showed a pronounced graded association with life satisfaction (*p* < 0.001). Physical activity and sleep duration (*p* < 0.001) were independently associated with higher SWLS. Being in a relationship or married was also associated with greater well-being.

**Conclusion:**

Health behaviors accounted for approximately half of the rural–urban gap in life satisfaction. The other half persisted independently, suggesting that structural disadvantage exerts effects on well-being that are not reducible to individual health practices. Campus interventions targeting sleep and physical activity are warranted, but closing the remainder of the gap will likely require strategies that address the cultural and institutional dimensions of rural-to-urban educational mobility.

## 1 Introduction

Graduate education in China’s elite universities offers an exceptional opportunity but also imposes intense psychological demands. Students navigate professional specialization, financial semi-dependence, identity consolidation, and often geographic dislocation within a compressed period, under the sustained pressure of publication deadlines, uncertain career prospects, and the cultural weight of family expectation (1–4). The mental health consequences of these pressures have been documented extensively. What has received far less attention is how the structural inequalities students carried with them before enrollment, in other words, the inequalities rooted in China’s persistent urban–rural divide, continue to shape their well-being once they are inside the gates of a top-tier institution.

For a student from a rural county or small town, admission to a Double First-Class university is a genuine achievement of educational mobility. But arrival does not erase the past. The transition can activate acculturative stress, for example, unfamiliar metropolitan environments, different social norms around leisure and networking, and often real financial constraint (5–7). Sociological research has documented the cultural mismatch, social isolation, and impostor syndrome that rural-origin students frequently experience (8–11). These are not transient adjustment difficulties; they reflect durable inequalities that the *hukou* system and decades of differential state investment have produced (8, 9). Poverty alleviation policies have raised absolute material conditions in rural China, but psychosocial and cultural gaps close more slowly (12–14).

At the same time, health behaviors, including sleep, physical activity, and substance use, are not distributed evenly across social groups. The Health Lifestyle Theory, as developed by Cockerham (15, 16), frames health practices as products of both structural conditions or life chances and individual agency or life choices. For a rural-origin graduate student, life chances may include having grown up with limited access to recreational sports facilities or with different norms around sleep and study. The choices that students make about exercise or sleep are then exercised within a new environment that may feel unsupportive (17, 18). Insomnia occupies a particularly consequential position, as it can be both a manifestation of distress and a driver of its worsening (3, 19, 20). Physical activity is among the most reliable behavioral correlates of mental health in student populations (19–23), yet it tends to be deprioritized under academic pressure.

The central gap this study addresses is not simply whether rural-origin students are less happy than their urban peers, as prior work already suggests they face greater challenges, but rather how much of any observed gap can be attributed to differential health behaviors, and how much persists independently of those behaviors. The distinction matters for intervention. If the gap is fully explained by health behaviors, the policy response is straightforward to improve sleep and increase exercise. If a substantial share remains after behavioral adjustment, then campus health strategies must also reckon with the harder-to-reach structural and cultural dimensions of rural-to-urban educational mobility.

We examined this question in a sample of graduate students at a major Double First-Class university in eastern China. We hypothesized: (1) Rural-origin students would report lower life satisfaction than urban-origin students, after adjustment for current financial resources. (2) Insomnia frequency would show a strong, graded negative association with life satisfaction. (3) Health behaviors would account for a meaningful portion of the rural–urban well-being gap.

## 2 Methods

### 2.1 Study design and participants

This cross-sectional study was prepared in accordance with the STROBE guidelines. Over 8 weeks (May 1–July 1, 2021), participants were recruited through WeChat, graduate association email lists, discipline-specific social media groups, and announcements circulated by the collaborating supervisor’s research group. The recruitment channels reached students primarily at a Double First-Class university. Eligible participants were master’s or doctoral students at the university. All provided written informed consent. The Ethics Committee in Shanghai Jiao Tong University approved the study protocol.

The required sample size was determined prior to data collection using two complementary approaches. First, for descriptive precision, the single-proportion formula SS = Z^2^ × P × (1 − P) / C^2^ was applied (24). Assuming *Z* = 1.96 (*α* = 0.05), *p* = 0.5 (maximal variance), and a desired precision of C = 5%, the minimum sample size was 385. Allowing for 10% non-response, the recruitment target was set at 428. Second, for the primary multivariable analysis, Green’s rule of thumb for linear regression (*N* ≥ 50 + 8 k) was applied (25). Anticipating up to 18 predictors in the fully adjusted model, the minimum required sample was 50 + 8 × 18 = 194. Both criteria were satisfied by the analytic sample, which was more than 428.

### 2.2 Measures

#### 2.2.1 Life satisfaction

The Satisfaction with Life Scale (SWLS) (26) uses five items (e.g., “In most ways my life is close to my ideal”) rated on a 7-point Likert scale (1 = Strongly Disagree, 7 = Strongly Agree), yielding total scores from 5 to 35. Cronbach’s alpha was 0.89 in this sample. The mean for Chinese adults in a nationally representative sample is approximately 23.0–24.5 (27); scores in the 20–24 range are regarded as average (26, 28).

#### 2.2.2 Physical activity

An instrument based on the International Physical Activity Questionnaire (IPAQ) short form (29, 30) captured frequency and duration of vigorous, moderate, and walking activity over the preceding 7 days. Following the standard IPAQ scoring protocol (31), fixed MET values were applied (vigorous = 8.0, moderate = 4.0, walking = 3.3 METs); the MET framework draws conceptually on the Compendium of Physical Activities (32). Data were cleaned per IPAQ truncation rules (daily bouts >180 min truncated to 180 min; weekly total >1,260 min truncated to 1,260 min). Total physical activity (Totalmetmink) was the sum of MET × minutes × days across the three intensity categories, expressed in MET-minutes per week. The variable has not been validated against accelerometry in this sample.

#### 2.2.3 Insomnia frequency

A single self-developed item asked about difficulty falling asleep, staying asleep, or early awakening in the past month. Responses included no, seldom (≤3 times/month), sometimes (1–2 times/month), often or daily (≥3 times/week).

#### 2.2.4 Hometown setting

A binary item to address the hometown setting. “Before entering university, was the location where you lived for the longest period best classified as: (1) Urban, or (2) Rural?”

#### 2.2.5 Covariates

We addressed the age (years), gender (Male/Female), ethnicity (Han/Minority), and marital/dating status. Monthly allowance, total disposable income from stipends, family support, and part-time work, was reported across six brackets (<1,000; 1,000–1,499; 1,500–1,999; 2,000–2,499; 2,500–2,999; ≥3,000 RMB). Average nightly sleep duration was collected with the open-ended item “On average, how many hours do you sleep per night?” Cigarette use and alcohol use were assessed.

### 2.3 Statistical analysis

Analyses used IBM SPSS 27.0. After descriptive summaries, univariate comparisons of SWLS across subgroups employed independent *t*-tests (dichotomous variables) or one-way ANOVA with Tukey *post-hoc* tests (multi-category variables). Pearson correlations were computed among continuous variables. Health behaviors significantly associated with SWLS were included in the regressions.

The primary analysis used hierarchical multiple linear regression with SWLS as the dependent variable. Two models were estimated. Model 1 entered sociodemographic variables only. Model 2 added the health behavior, including sleep time, Totalmetmink, insomnia, and alcohol use. This structure makes it possible to isolate the incremental contribution of health behaviors, as the change in the hometown coefficient across models estimates the share of the rural–urban gap.

For both models, the following diagnostic checks were performed: (a) normality of residuals was assessed via histogram and P–P plot inspection; (b) homoscedasticity was evaluated by plotting standardized residuals against standardized predicted values; (c) multicollinearity was examined using variance inflation factors (VIF); (d) the Durbin–Watson statistic was examined to assess independence of residuals. The distribution of Totalmetmink was inspected for skewness. We reported standardized (*β*) coefficients, standard errors (SE), *p*-values, adjusted R^2^, and model F-statistics.

## 3 Results

### 3.1 Sample characteristics

We received 523 questionnaires. After excluding submissions with >10% missing data on core variables or logically inconsistent response patterns, 497 valid responses were retained. Table 1 summarizes the 497 participants. Mean age was 25.01 (SD = 3.27); 66.6% were male; 93.96% were Han Chinese. A quarter of the sample (25.15%) reported a rural hometown. The most common monthly allowance bracket was 1,500–1,999 RMB (24.75%). In terms of relationships, 43.46% were neither dating nor married, 47.69% were dating.

**Table 1:** Sample characteristics.

Characteristic	*N*	%
Gender		
Male	331	66.60
Female	166	33.40
Hometown setting		
Urban	372	74.85
Rural	125	25.15
Ethnicity		
Han Chinese	467	93.96
Minority nationality	30	6.04
Monthly allowance (RMB)		
<1,000	16	3.22
1,000–1,499	84	16.90
1,500–1,999	123	24.75
2,000–2,499	117	23.54
2,500–2,999	47	9.46
≥3,000	110	22.13
Marital/dating status		
Not dating nor married	216	43.46
Dating but unmarried	237	47.69
Married	41	8.25
Others	3	0.60
Insomnia frequency		
No	160	32.19
Seldom (≤3 times/month)	191	38.43
Sometimes (1–2)	115	23.14
Often or Daily (≥3 times/ month)	31	6.24
Cigarette use		
Never smoked	482	96.98
Ever smoked	15	3.02
Alcohol use		
Never	260	52.31
Rarely (≤2 times/month)	198	39.84
Sometimes (≤4 times/month)	30	6.04
Often or Always (≥12 times/month)	9	1.81
	Mean	SD
Age (years)	25.01	3.27
Total metmink (MET-min/week)	3,000.70	520.75
SWLS total score	23.29	6.23
Sleep time (hours/night)	7.62	1.98

Sleep data were concerning. Only a third (32.19%) reported no insomnia; 38.43% experienced symptoms seldom, 23.14% sometimes, and 6.24% often or daily. Most participants were non-smokers (96.98%), and 52.31% reported no alcohol consumption in the past month.

Mean SWLS was 23.29 (SD = 6.23, range 5–35), in line with norms reported for Chinese adults (27). Mean Totalmetmink was 3,000.70 MET-min/week (SD = 520.75); the median of 2,847 indicated right skew. Mean sleep duration was 7.62 h (SD = 1.98); roughly 28% averaged less than 7 h.

### 3.2 Univariate associations with life satisfaction

Table 2 presents SWLS scores across participant subgroups. The rural–urban gap was clear, as rural-origin students scored 21.72 versus 23.82 for urban-origin students (*p* = 0.001, *d* = 0.34). Women scored modestly higher than men (24.43 vs. 22.72, *p* = 0.004, *d* = 0.27). Monthly allowance and ethnicity were not associated with SWLS (all *p* > 0.05).

**Table 2:** Univariate associations with life satisfaction (SWLS).

Variable	SWLS M ± SD	t/F	*p*
Gender		−2.92	**0.004**
Male	22.72 ± 6.25		
Female	24.43 ± 6.05		
Hometown setting		3.29	**0.001**
Urban	23.82 ± 6.12		
Rural	21.72 ± 6.33		
Ethnicity		1.63	0.105
Han Chinese	23.41 ± 6.12		
Minority nationality	21.50 ± 7.65		
Monthly allowance (RMB)		0.86	0.511
<1,000	22.69 ± 7.27		
1,000–1,499	22.39 ± 6.55		
1,500–1,999	22.94 ± 5.93		
2,000–2,499	23.68 ± 5.31		
2,500–2,999	23.34 ± 5.91		
≥3,000	24.03 ± 7.15		
Marital/dating status		3.51	**0.015**
Not dating nor married	22.30 ± 6.43		
Dating but unmarried	23.93 ± 5.95		
Married	24.56 ± 6.23		
Insomnia frequency		14.48	**<0.001**
No	25.64 ± 5.77		
Seldom (≤3 times/month)	22.87 ± 5.74		
Sometimes (1–2)	22.09 ± 6.18		
Often or Daily (≥3 times/week)	19.42 ± 6.20		
Cigarette use		0.80	0.451
Never smoked	23.30 ± 6.18		
Ever smoked	22.54 ± 7.86		
Alcohol use		2.98	**0.019**
Never	23.28 ± 6.36		
Rarely (≤2 times/month)	23.33 ± 5.99		
Sometimes (≤4 times/month)	24.07 ± 5.53		
Often or Always (≥12 times/month)	16.11 ± 9.86		

Bold: *p* < 0.05.

Insomnia displayed the strongest univariate signal. SWLS dropped from 25.64 (No insomnia) to 19.42 (Often or Daily), *F* = 14.48, *p* < 0.001. Marital status was also associated with SWLS (*p* = 0.015).

### 3.3 Bivariate correlations

Table 3 displays Pearson correlations. Sleep duration correlated positively with both SWLS and Totalmetmink (both *r* = 0.198, *p* < 0.001). The strongest bivariate correlation was between Totalmetmink and SWLS (*r* = 0.546, *p* < 0.001). Age showed a weak negative association with SWLS (*r* = −0.115, *p* = 0.010).

**Table 3:** Pearson correlations.

	Age	Sleeptime	Totalmetmink	SWLS
Age	1			
Sleeptime	0.077	1		
Totalmetmink	0.022	0.198***	1	
SWLS	−0.115*	0.198***	0.546***	1

***p < 0.001, *p < 0.05.

### 3.4 How much of the rural–urban gap do health behaviors account for?

Table 4 presents the hierarchical regression models. In Model 1 (sociodemographic variables only), rural origin was associated with a reduction in SWLS (*β* = −0.133, *p* = 0.003). After adding sleep duration, physical activity, insomnia, and alcohol use in Model 2, the rural-origin coefficient reduced 49% roughly (*β* = −0.065, *p* = 0.088). The residual coefficient was marginally significant.

**Table 4:** Hierarchical regression models for life satisfaction (SWLS).

	Model 1			Model 2		
	*β*	SE	*p*	*β*	SE	*p*
Sociodemographic						
Hometown (ref: urban)	−0.133	0.658	0.003	−0.065	0.585	0.088
Age (years)	−0.152	0.088	0.001	−0.164	0.074	<0.001
Gender (ref: male)	0.107	0.607	0.018	0.068	0.517	0.074
Monthly allowance (ref: ≥3,000 RMB)						
<1,000	−0.032	1.582	0.476	−0.017	1.321	0.643
1,000–1,499	−0.104	0.901	0.061	−0.062	0.754	0.181
1,500–1,999	−0.076	0.827	0.189	−0.034	0.695	0.484
2,000–2,499	−0.019	0.822	0.733	0.001	0.698	0.981
2,500–2,999	−0.025	1.048	0.615	−0.018	0.864	0.653
Marital status (ref: Not dating)						
Dating but unmarried	0.152	0.574	0.001	0.116	0.476	0.002
Married	0.169	1.068	<0.001	0.124	0.913	0.002
Health behaviors						
Sleep time (hours)				0.177	0.118	<0.001
Totalmetmink (per MET-min/week)				0.484	0.001	<0.001
Insomnia (ref: No insomnia)						
Seldom (≤3/month)				−0.174	0.649	<0.001
Sometimes (1–2)				−0.218	0.757	<0.001
Often or Daily (≥3/ month)				−0.191	1.334	<0.001
Alcohol Use (ref: Never)						
Rarely (≤2/month)				0.026	0.500	0.520
Sometimes (≤4/month)				−0.007	0.982	0.848
Often or Always (≥12/month)				0.004	1.890	0.927
Model Fit						
Adjusted R^2^	0.080			0.421		
ΔR^2^	—			0.341		
F	7.89***			13.76***		

***p < 0.001.

This attenuation is the core empirical finding. Health behaviors and sleep accounted for about half of the rural–urban gap. The other half persisted independently, as after equalizing sleep, activity, and insomnia in the model, rural-origin students were still lower than their urban-origin peers.

Model diagnostics for the regression analyses in Table 4 were satisfactory. All variance inflation factors were below 2.5 (highest: 2.31 for sleeptime), indicating no problematic multicollinearity. The Durbin–Watson statistic was 1.94, within the acceptable range for independence of residuals. Visual inspection of histograms and normal P–P plots confirmed approximate normality of residuals, and plots of standardized residuals against fitted values showed no evidence of heteroscedasticity. Totalmetmink exhibited mild positive skew (skewness = 1.12); sensitivity analyses using log-transformed values yielded substantively identical results, supporting the use of untransformed values in the reported models.

## 4 Discussion

There were two key findings in this research. First, rural-origin graduate students reported lower life satisfaction than their urban-origin peers, and roughly half of this gap was associated with differences in sleep, physical activity, and insomnia. Second, the other half of the gap persisted after adjusting for these behavioral variables.

### 4.1 What health behaviors explain—and what they do not

The hierarchical models tell a clear story. In Model 1, rural origin carried a negative coefficient (*p* = 0.003). Adding sleep, physical activity, and insomnia cut that coefficient nearly in half (*p* = 0.088). That 49% attenuation is the share of the rural–urban gap associated with differential health behaviors. It means that if rural and urban students slept the same amount, moved the same amount, and experienced the same insomnia frequency, the well-being gap between them would be half closed.

The behavioral attenuation also points to a pathway. Rural-origin students had lower unadjusted physical activity than urban-origin students, and physical activity was the strongest behavioral correlate of life satisfaction. The pattern fits a model in which structural background constrains health behaviors, and those behaviors in turn matter for how students feel. Formal mediation analysis with bootstrapped confidence intervals would be needed to establish the indirect effects we are describing in suggestive terms.

### 4.2 The residual half: structural disadvantage beyond behavior

If health behaviors account for roughly half the gap, what explains the rest? The mechanisms we would need to answer the question were not in our dataset, for example, acculturative stress, perceived social distance, institutional belonging, and differential access to the informal networks that facilitate academic and professional advancement (10–14, 33). But the regression result narrows the possibilities. Whatever produces the residual gap, it is not fully captured by sleep, physical activity, insomnia, or current monthly income.

Conceptually, it suggests that rural origin functions as more than a demographic correlate of poor health behaviors. The persistence of the SWLS gap after behavioral equalization is consistent with the view that hometown setting represents a bundle of exposures, including economic, cultural, social, and psychosocial, that operate through channels beyond individual health practices. This aligns with a growing body of work in social epidemiology demonstrating that place-based disadvantage leaves a durable imprint on well-being that is not fully mediated by downstream behavioral pathways (27, 32). In the Chinese context, the *hukou* system, differential educational investment, and the cultural coding of rural identity as lower status create conditions in which rural-origin students, even after gaining admission to an elite institution, continue to navigate an environment in which they are a numerical and cultural minority. The strain this produces, what Chen (9) terms “hysteresis effects” and Xie (8) describes as “misrecognition” of cultural capital, may operate as a persistent, low-grade stressor that is not alleviated by sleeping more or exercising more.

Practically, this means that behavioral interventions, while important, are insufficient on their own. Sleep hygiene campaigns and physical activity programs target the mechanisms that account for roughly half the gap. The other half requires strategies that engage with the social and institutional fabric of the university, including mentoring structures that pair rural-origin students with faculty or senior students who share their background; community-building initiatives that bridge rather than reinforce social distance between urban and rural students; and institutional recognition that the transition from a rural hometown to an elite urban campus is a genuine cultural shift, not merely a geographic one. These interventions are harder to design, implement, and evaluate than a sleep hygiene campaign. But the data argue that they are not optional. A university that is serious about health equity cannot treat the rural–urban well-being gap as a problem that will be solved by sleep apps.

What the data do show is that the gap is real, that it is large enough to matter, and that about half of it is not accounted for by the behavioral variables that dominate the current campus health conversation. That is a warrant for broadening the conversation.

### 4.3 Other behavioral correlates

Several other patterns are worth noting briefly. Insomnia showed the strongest and most clearly graded association with SWLS. CBT-I has a strong evidence base for treating insomnia in student populations (34). Physical activity and sleep duration were each independently associated with life satisfaction. Social connection, operationalized as dating or marital status, was a consistent independent correlate, but graduate school is not an environment that naturally supports relationship formation (35–38). The female advantage in SWLS was attenuated to non-significance after behavioral adjustment, consistent with prior work showing that gender differences in well-being among Chinese students partly reflect differential exercise and sleep patterns (39). Age showed a persistent inverse association with SWLS, likely reflecting the accumulated strain of extended doctoral timelines (40, 41). Together, these patterns reinforce the point that health behaviors are strongly linked to life satisfaction in this population.

### 4.4 Limitations

Several limitations should be considered. First, the cross-sectional design precludes causal inference. Reverse causation and unmeasured confounding remain plausible. The hierarchical regression partitions variance but does not establish mediation or causal ordering. Second, the sample was drawn from a single university in Shanghai through convenience channels. Self-selection bias is likely, and the findings do not generalize to the national Double First-Class graduate population. Third, key variables were measured with single items or self-reports, for example, hometown origin (one binary item), sleep, and insomnia. All variables came from the same questionnaire, introducing potential common method variance. Fourth, although the sample met prospective size requirements, post-hoc assessment indicates it was underpowered to detect small independent effects (f^2^ ≈ 0.006). The residual rural-origin coefficient would require approximately 1,300 participants for 80% power at *α* = 0.05, and its marginal *p*-value should be interpreted with this constraint in mind. Fifth, variable selection combined univariate screening with theoretical judgment rather than a single pre-specified rule. No multiplicity correction was applied to univariate comparisons, and no formal mediation analysis was conducted.

### 4.5 Implications for campus public health

Two messages follow for university health promotion. Sleep, physical activity, and social connection are the behavioral domains most strongly associated with graduate student life satisfaction in this sample, and they are sensible targets for rigorous intervention trials. At the same time, the persistence of a rural–urban gap after behavioral adjustment signals that campuses committed to health equity must look beyond individual-level health promotion. Structural investments, including community-building across social distances and institutional recognition of the cultural dimensions of rural-to-urban educational mobility, warrant serious attention alongside behavioral programs (42–46).

## 5 Conclusion

In this cross-sectional study of 497 graduate students at an elite Chinese university, rural-origin students reported lower life satisfaction than urban-origin students. Differences in sleep, physical activity, and insomnia accounted for roughly half of this gap. The other half persisted independently, which meant that behavioral equalization alone would not eliminate. The full behavioral model explained 42% of the variance in SWLS. These results make a case for investing in campus sleep and physical activity programs. But they also suggest that closing the rural–urban well-being gap will take more than behavioral interventions. The cultural and institutional dimensions of educational mobility need attention, too.

## Data Availability

The original contributions presented in the study are included in the article/supplementary material, further inquiries can be directed to the corresponding author.

## References

[1] Satinsky EN Kimura T Kiang MV Abebe R Cunningham S Lee H . Systematic review and meta-analysis of depression, anxiety, and suicidal ideation among Ph.D. students. Sci Rep. (2021) 11:14370. doi: 10.1038/s41598-021-93687-7, 34257319 PMC8277873

[2] Eisenberg D Gollust SE Golberstein E Hefner JL. Prevalence and correlates of depression, anxiety, and suicidality among university students. Am J Orthopsychiatry. (2007) 77:534–42. doi: 10.1037/0002-9432.77.4.534, 18194033

[3] Lund HG Reider BD Whiting AB Prichard JR. Sleep patterns and predictors of disturbed sleep in a large population of college students. J Adolesc Health. (2010) 46:124–32. doi: 10.1016/j.jadohealth.2009.06.016, 20113918

[4] Yu GL Wang XZ. Basic conditions and educational countermeasures of mental health problems in Chinese graduate students. China High Educ Res. (2024) 40:80–7. doi: 10.16298/j.cnki.1004-3667.2024.07.12

[5] Wang S Chen J Zhou L. After admission: the emotional suffering of students enrolled through the rural students quota plan in China’s elite universities. Eur J Educ. (2024) 59:e12774. doi: 10.1111/ejed.12774

[6] Long Y Jia C Luo X Sun Y Zuo W Wu Y . The impact of higher education on health literacy: a comparative study between urban and rural China. Sustainability. (2022) 14:12142. doi: 10.3390/su141912142

[7] Short SE Mollborn S. Social determinants and health behaviors: conceptual frames and empirical advances. Curr Opin Psychol. (2015) 5:78–84. doi: 10.1016/j.copsyc.2015.05.002, 26213711 PMC4511598

[8] Xie A. Desirability, technical skills, and misrecognition: cultural capital and rural students’ social integration in elite Chinese universities. Poetics. (2022) 92:101645. doi: 10.1016/j.poetic.2022.101645

[9] Chen Y. Hysteresis effects and emotional suffering: Chinese rural students’ first encounters with the urban university. Sociol Res Online. (2022) 27:101–17. doi: 10.1177/1360780420949884

[10] Li RH Wang Y Zhang L Chen X. A systematic review on the impact of social support on college students’ wellbeing and mental health. PLoS One. (2025) 20:e0325212. doi: 10.1371/journal.pone.0325212 40644433 PMC12250717

[11] Li W Cui E. Psychological strain among Chinese master’s students: protective mechanisms of self-esteem and influences of urban-rural background and gender. Front Psychol. (2026) 17:1729754. doi: 10.3389/fpsyg.2026.1729754, 41797999 PMC12963288

[12] Mulvey B Li B. The negotiation of socio-spatial background among elite Chinese university graduates. Popul Space Place. (2025) 31:e70055. doi: 10.1002/psp.70055

[13] Wong YL Liao Q. Cultural capital and habitus in the field of higher education: academic and social adaptation of rural students in four elite universities in Shanghai, China. Camb J Educ. (2022) 52:775–93. doi: 10.1080/0305764X.2022.2056142

[14] Yang J Islem S McNamee TC. Rural students’ sense of belonging: the influence of academic campus climate. New Dir High Educ. (2025) 2025:47–56. doi: 10.1002/he.20526

[15] Cockerham WC. Health lifestyle theory and the convergence of agency and structure. J Health Soc Behav. (2005) 46:51–67. doi: 10.1177/002214650504600105, 15869120

[16] Brivio F Vigano A Paterna A Palena N Greco A. Narrative review and analysis of the use of “lifestyle” in health psychology. Int J Environ Res Public Health. (2023) 20:4427. doi: 10.3390/ijerph20054427, 36901437 PMC10001804

[17] Gao Y Li X Zhang H. Future self-continuity predicts health-promoting behaviors of Chinese rural college students: evidence from mental imagery intervention. J Psychol. (2024) 158:179–99. doi: 10.1080/00223980.2023.2279532, 38181206

[18] Benítez-Agudelo JC Restrepo D Navarro-Jimenez E Clemente-Suárez VJ. Longitudinal effects of stress in an academic context on psychological well-being, physiological markers, health behaviors, and academic performance in university students. BMC Psychol. (2025) 13:753. doi: 10.1186/s40359-025-02678-2 40629456 PMC12239435

[19] Li B Jiang W Han SS Ye YP Li YX Lou H . Influence of moderate-to-high intensity physical activity on depression levels: a study based on a health survey of Chinese university students. BMC Public Health. (2024) 24:1023. doi: 10.1186/s12889-024-18433-w, 38609890 PMC11015539

[20] Li B Han SS Ye YP Li YX Meng SQ Feng S . Cross-sectional associations of physical activity and sleep with mental health among Chinese university students. Sci Rep. (2024) 14:31045. doi: 10.1038/s41598-024-80034-9, 39738254 PMC11686290

[21] Schuch FB Vancampfort D Firth J Rosenbaum S Ward PB Silva ES . Physical activity and incident depression: a meta-analysis of prospective cohort studies. Am J Psychiatry. (2018) 175:631–48. doi: 10.1176/appi.ajp.2018.17111194, 29690792

[22] Chekroud SR Gueorguieva R Zheutlin AB Paulus M Krumholz HM Krystal JH . Association between physical exercise and mental health in 1.2 million individuals in the USA between 2011 and 2015: a cross-sectional study. Lancet Psychiatry. (2018) 5:739–46. doi: 10.1016/S2215-0366(18)30227-X, 30099000

[23] Gao XL Zhang JH Yang Y Cao ZB. Sedentary behavior, screen time and mental health of college students: a meta-analysis. Zhonghua Liu Xing Bing Xue Za Zhi. (2023) 44:477–85. doi: 10.3760/cma.j.cn112338-20220728-00669, [in Chinese] 36942345

[24] Naing L Nordin RB Abdul Rahman H Naing YT. Sample size calculation for prevalence studies using Scalex and ScalaR calculators. BMC Med Res Methodol. (2022) 22:209. doi: 10.1186/s12874-022-01694-7, 35907796 PMC9338613

[25] Green SB. How many subjects does it take to do a regression analysis? Multivariate Behav Res. (1991) 26:499–510. doi: 10.1207/s15327906mbr2603_7, 26776715

[26] Diener E Emmons RA Larsen RJ Griffin S. The satisfaction with life scale. J Pers Assess. (1985) 49:71–5. doi: 10.1207/s15327752jpa4901_13 16367493

[27] Bai X Wu C Zheng R Ren X. The psychometric evaluation of the satisfaction with life scale using a nationally representative sample of China. J Happiness Stud. (2011) 12:183–97. doi: 10.1007/s10902-010-9186-x

[28] Checa I Perales J Espejo B. Measurement invariance of the satisfaction with life scale by gender, age, marital status and educational level. Qual Life Res. (2019) 28:963–8. doi: 10.1007/s11136-018-2066-2, 30484120 PMC6440802

[29] Qu NN Li KJ. Study on the reliability and validity of international physical activity questionnaire (Chinese version, IPAQ). Chin J Epidemiol. (2004) 25:265–8. [in Chinese]. doi: 10.3760/j.issn:0254-6450.2004.03.021 15200945

[30] Fan MY Lyu J He PP. Chinese guidelines for data processing and analysis concerning the international physical activity questionnaire. Chin J Epidemiol. (2014) 35:961–4. doi: 10.3760/cma.j.issn.0254-6450.2014.08.019, [in Chinese] 25376692

[31] Craig CL Marshall AL Sjöström M Bauman AE Booth ML Ainsworth BE . International physical activity questionnaire: 12-country reliability and validity. Med Sci Sports Exerc. (2003) 35:1381–95. doi: 10.1249/01.MSS.0000078924.61453.FB 12900694

[32] Ainsworth BE Haskell WL Herrmann SD Meckes N Bassett DR Jr Tudor-Locke C . 2011 compendium of physical activities: a second update of codes and MET values. Med Sci Sports Exerc. (2011) 43:1575–81. doi: 10.1249/MSS.0b013e31821ece12 21681120

[33] Xie A Kuang H Hong Y. Favouring middle- and upper-class students? The structure and process of attending China’s selective universities. Cent Eur J Educ Res. (2023) 5:1–16. doi: 10.37441/cejer/2023/5/1/12393

[34] Ghoul Z Pernice FM Branson JS Lumley MA. The impact of a remote, brief, sleep hygiene intervention on undergraduate students’ sleep and stress: a randomized controlled trial. J Am Coll Heal. (2025) 73:3976–84. doi: 10.1080/07448481.2025.2464762, 40020151

[35] Cummins RA. "Measuring happiness and subjective well-being". In: David SA Boniwell I Conley Ayers A, editors. Oxford Handbook of Happiness. Oxford: Oxford University Press (2013). p. 185–200.

[36] Diener E Emmons RA. The independence of positive and negative affect. J Pers Soc Psychol. (1984) 47:1105–17. doi: 10.1037/0022-3514.47.5.1105, 6520704

[37] Emerson SD Guhn M Gadermann AM. Measurement invariance of the satisfaction with life scale: reviewing three decades of research. Qual Life Res. (2017) 26:2251–64. doi: 10.1007/s11136-017-1552-2, 28324322

[38] Petruzziello G Soncini A Puzzo G Tomei G Toscano F. The Psychological and Professional Well-Being of Doctoral Students: Insights from Two Cohorts of a Major Italian University. Advances in Psychology Research, (2024) 153:1–25.

[39] Hao M Liu X Wang Y Wu Q Yan W Hao Y. The associations between body dissatisfaction, exercise intensity, sleep quality, and depression in university students in southern China. Front Psych. (2023) 14:1118855. doi: 10.3389/fpsyt.2023.1118855, 37020733 PMC10067572

[40] Casey C Harvey O Taylor J Knight F Trenoweth S. Exploring the wellbeing and resilience of postgraduate researchers. J Further High Educ. (2022) 46:850–67. doi: 10.1080/0309877X.2021.2018413

[41] He A Mu W. Under the shadow of age: a moderated mediation model of academic stress, burnout, self-criticism, and depression among Chinese PhD students. High Educ. (2025). doi: 10.1007/s10734-025-01539-4

[42] Hall BJ Xiong P Guo X Sou EKL Chou UI Shen Z. An evaluation of a low intensity mHealth enhanced mindfulness intervention for Chinese university students: a randomized controlled trial. Psychiatry Res. (2018) 270:394–403. doi: 10.1016/j.psychres.2018.09.060, 30300870

[43] Nair B Otaki F. Promoting university students’ mental health: a systematic literature review introducing the 4M-model of individual-level interventions. Front Public Health. (2021) 9:699030. doi: 10.3389/fpubh.2021.699030, 34249852 PMC8267876

[44] Worsley JD Pennington A Corcoran R. Supporting mental health and wellbeing of university and college students: a systematic review of review-level evidence of interventions. PLoS One. (2022) 17:e0266725. doi: 10.1371/journal.pone.0266725, 35905058 PMC9337666

[45] Malagodi F Findon J Gardner B Dommett E. A systematic review of the effectiveness of physical activity interventions for improving mental health and wellbeing in university students. J Coll Stud Ment Health. (2025). 1–37. doi: 10.1080/28367138.2025.2566914

[46] Yu Y Liang Z Zhou Q Tuersun Y Liu S Wang C . Decomposition and comparative analysis of urban-rural disparities in eHealth literacy among Chinese university students: cross-sectional study. J Med Internet Res. (2025) 27:e63671. doi: 10.2196/63671, 40138681 PMC11982776

